# Superplastic Deformation Behavior and Microstructural Evolution of Electroformed Nickel Foils Determined by Thermomechanical Analysis

**DOI:** 10.3390/ma18061365

**Published:** 2025-03-19

**Authors:** Minsu Lee, Hohyeong Kim, Jinho Ahn

**Affiliations:** 1 Industrial Components R&D Department, Korea Institute of Industrial Technology, Incheon 21999, Republic of Korea; lms0120@kitech.re.kr; 2 Department of Materials Science and Engineering, Hanyang University, Seoul 04763, Republic of Korea; 3 Department of Materials Science and Engineering, Inha University, Incheon 22212, Republic of Korea

**Keywords:** superplasticity, thermomechanical analysis, electrodeposition, nanocrystalline nickel, microstructure

## Abstract

Superplastic deformation, which occurs when fine-grained metals exhibit high ductility (often exceeding 300%) under specific conditions at approximately half of their melting temperature, allows the creation of complex shapes required by the aerospace and electronic material industries. Typically, superplastic characteristics are evaluated using universal testing machines (UTMs). However, nickel (Ni) and its alloys, which are applied as electrodeposits in the fabrication of electronic materials, are nanocrystalline in nature and exhibit superplasticity under specific temperatures and deformation conditions. Electrodeposited foils are very thin, making traditional UTM testing challenging; therefore, a new approach is required. In this study, we used a thermomechanical analyzer (TMA) to analyze the superplastic properties of electrodeposited nickel foils simply and precisely. TMAs are particularly appropriate when evaluating thin foils because they yield detailed thermal deformation data, whereas UTMs do not. A TMA reveals thermal deformation of electrodeposited nickel foils across various temperatures, as well as microstructures and grain growth. We performed superplastic analysis at 400 °C, 500 °C, and 600 °C at a strain rate of 1 × 10^−3^ s^−1^, and microstructural data were obtained through X-ray diffraction and electron backscatter diffraction. Superplastic deformation was apparent at 400 °C. The data obtained through our systematic analysis using a TMA will guide future studies on the application of superplastic properties of electrodeposited nanocrystalline nickel foils.

## 1. Introduction

Superplasticity occurs when a material exhibits elongation greater than 300%; under these conditions, fine grains must be maintained at appropriate formation temperatures and strain rates [1,2,3]. When the grain size remains below 10 μm over specific temperature ranges at low deformation rates, the material is considered superplastic [4,5]. Superplastic materials can form complex shapes under minimal applied force; extensive research has been conducted on superplasticity in aluminum (Al), titanium (Ti), copper (Cu), iron (Fe), magnesium (Mg), and their alloys [6,7,8]. Superplasticity can develop when the grain size of a bulk material is reduced to below a few micrometers and specific strain is then applied. This phenomenon has been exploited by the aerospace industry, which employs lightweight, corrosion-resistant, high-strength Al and Ti alloys [9,10,11]. Superplastic deformation is generally considered to include grain boundary sliding, dislocation, and diffusion flow [12,13,14]. Recent studies have revealed that dynamic recrystallization serves as the primary deformation mechanism of Ni-based and Ni-Co-based superalloys, in addition to conventional deformation mechanisms [15,16,17]. However, it is difficult to refine the grain size of bulk materials because material costs increase. Recent research on superplastic deformation has sought higher formation speeds at lower temperatures [18,19,20,21]. Electrodeposited metals exhibit nanocrystalline structures; on appropriate heating treatment, grains can grow to the micrometer level [22,23,24,25], allowing the manufacture of metals with excellent superplastic properties. Electrodeposited nickel (Ni) and Ni alloys exhibit superplastic characteristics at 350–500 °C, depending on the plating conditions [26,27,28,29]. Sulfur-containing saccharin is commonly added during nickel plating to suppress internal stress and promote grain refinement [30,31]. Superplastic deformation of electrodeposited nickel is influenced by sulfur atoms at grain boundaries, causing the boundaries to slide [32,33]. Various foils comprising Ni and Ni alloys exhibit excellent mechanical properties and good thermal stability. These superplastic characteristics are applied in batteries, electronic materials, and small electronic components [34,35,36].

The nanocrystalline structure created during electroforming initially features fine grains. However, the grains may then grow rapidly during thermal treatment or deformation, rendering it difficult to maintain superplastic behavior. Thin electrodeposited metal foils have certain limitations when using conventional testing methods to evaluate superplastic behavior. Therefore, a new analytical method is required. Previous studies evaluated electrodeposited foils thicker than 100 µm employing universal testing machines (UTMs) [24,32,33]. However, the thickness and microstructure of metal foils depend on the electrodeposition process parameters. With increasing thickness, a multi-scale hierarchical structure has been observed to develop [37,38]. This means that metal foils of micrometer-scale thicknesses may exhibit behaviors distinct from those of conventional superplasticity. Moreover, grip issues arising during UTM-based superplastic testing and difficulties encountered when preparing thin metal sheet specimens limit evaluation to foils no more than tens of micrometers thick. Accurate evaluation of superplasticity is essential because this greatly affects superplastic formation [39,40,41]. Simple analytical methods are required to establish the optimal conditions under which various superplastic alloys form. Recently, Ni and Ni alloy foils fabricated via electroforming have garnered attention in terms of industrial applications. Such foils serve as stencils during the fabrication of flip-chip ball grid array (FCBGA) packages and fine metal masks (FMMs) for OLEDs. The foils facilitate micro-pattern formation and precise hole design, which cannot be achieved using conventional rolled materials [42,43,44]. Moreover, optimizing the mechanical and thermal properties of such ultrathin foils (≤10 μm) is crucial, as their reduced thickness significantly affects these characteristics. Understanding these properties is essential for ensuring the reliability of ultrathin foils in high-precision applications, including next-generation electronics and flexible devices.

In this study, we analyzed the superplastic electrodeposited Ni foils with a thickness of 10 μm and assessed their industrial utility. We used a thermomechanical analyzer (TMA) rather than a traditional uniform testing machine (UTM) to analyze the thermal behaviors and superplastic deformation of electrodeposited, nanocrystalline Ni foils. This novel approach allowed us to evaluate precisely the micro-thermal deformation of thin metals, as well as microstructural changes and grain evolution under different annealing temperatures. Our results demonstrate the value of the TMA as a simple tool for analyzing the superplastic behaviors of thin metal foils.

## 2. Experimental

### 2.1. Electroforming of Nanocrystalline Ni Foils

**Figure 1:**
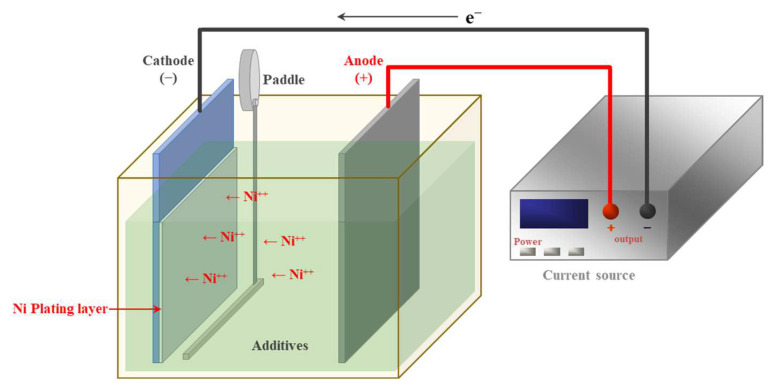
Schematic of paddle agitation electrodeposition.

Nanocrystalline Ni foils were fabricated via electrodeposition in a paddle-agitated plating bath (Figure 1). The metal salts used were Ni(II) sulfamate tetrahydrate (180 g/L) and Ni(II) chloride hexahydrate (20 g/L). The additives were boric acid (30 g/L), saccharin (1 g/L), and sodium dodecyl sulfate (0.1 g/L). The bath temperature was 60 °C, and the current density was 60 mA/cm^2^. The foil growth rate was approximately 1 μm/min, and the final thickness was 10 μm. The anode was composed of Ni S-Rounds (99.11% pure), and the cathode was composed of grade 304L stainless steel. The dimensions of both the anode and cathode were 100 mm × 100 mm (1:1 ratio). During plating, the pH was maintained at 4, and the paddle agitation speed was maintained at 10 m/s. The electrodeposition conditions are summarized in Table 1. After electrodeposition, the foils were separated from the stainless steel, and a fiber laser (LG-20P, Ideal Laser, Guangzhou, China) was used to prepare samples for superplastic analysis. The specimens were prepared as flat plates with a 4 mm width for the thermal experiments and a 1 mm width for the superplastic analyses. All plates were 10 μm in thickness.

### 2.2. Thermal Behavior and Superplasticity Analysis

Glass transition temperature (Tg) differential scanning calorimetry (DSC) was conducted using the LABSYS evo thermal analysis platform (SETARAM Instrumentation, Caluire, France) with a temperature gradient from room temperature to 1100 °C at a heating rate of 5 °C/min. TMA data were collected from room temperature to 800 °C at a heating rate of 5 °C/min. For TMA analysis, each foil was secured using an upper/lower clamp designed for film samples and mounted on the Film/Fiber probe. An X-ray diffraction (XRD) device (MiniFlex II, Rigaku, Tokyo, Japan) was used to assess the crystal structure and nanocrystalline grain size. Electrodeposited Ni foils were heat-treated in hydrogen at 400 °C, 500 °C, and 600 °C, and a TMA (TMA-Q400EM, TA Instruments, New Castle, DE, USA) was used to compare the data with those of bulk samples subjected to superplastic experiments at the same temperatures. During superplastic analysis using the TMA, each sample was mounted on a stage under nitrogen delivered at 100 mL/min. Superplastic tensile tests were performed using a TMA film/fiber probe operating in the advanced mode. The sample width and thickness were input, the lengths were measured before heating, and the temperature and strain rate were maintained at specific values. The temperature was increased from room temperature at 5 °C/min and maintained for 30 min at 400 °C, 500 °C, or 600 °C. Deformation proceeded at a strain rate of 1 × 10^−3^ s^−1^ at these temperatures. To observe microstructures, samples were prepared for electron backscatter diffraction (EBSD) analysis using a focused ion beam (Nova NanoLab 600, FEI, Hillsboro, OR, USA). Grain evolution and size distribution were analyzed. The EBSD analysis featured a scan step size of 0.25 μm. The grain misorientation threshold was 2°. Grain boundaries were classified as low-angle (2–15°) or high-angle (>15°). The proportions of such boundaries were approximately 2% and 98%, respectively.

## 3. Results and Discussion

### 3.1. Thermal Behavior Analysis

**Figure 2:**
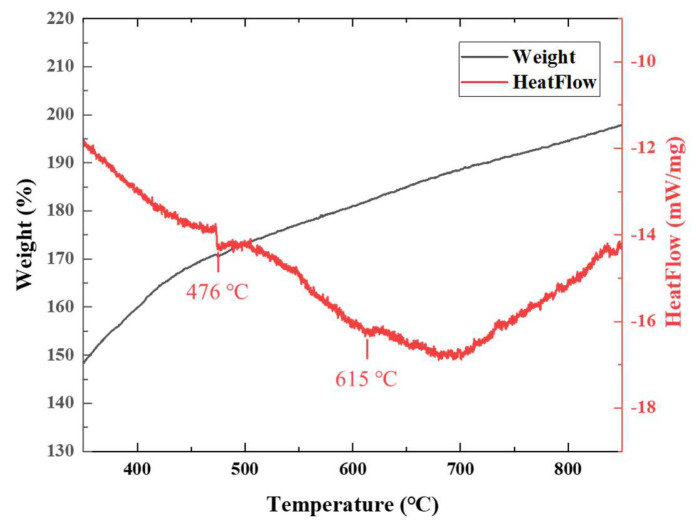
The TG-DSC curves of an electrodeposited Ni foil at a heating rate of 5 °C/min.

The TG-DSC data for electrodeposited nanocrystalline Ni foils are shown in Figure 2. We sought to predict phase transitions and the temperature range at which superplasticity might develop. Figure 2 shows a mass increase with the rise in temperature, probably attributable to the oxidation of nanocrystalline Ni, as the nitrogen atmosphere may have contained minute amounts of oxygen or other impurities. The DSC curve shows exothermic peaks at approximately 476 °C and 615 °C, reflecting heat release during sudden phase transitions. The peak positions varied slightly under the influence of small, variable residual amounts of additives used during electrodeposition. However, in previous studies, such peaks were taken to indicate the temperature range over which nanocrystalline structures were transformed into more stable, fully crystalline structures. The thermal transition observed at 476 °C is attributed to a glass transition-like phenomenon, likely associated with the structural rearrangement of grain boundaries in this nanocrystalline nickel material. Accordingly, the glass transition temperature (Tg) of this sample is estimated to be approximately 476 °C. As the temperature increases, grain boundary relaxation occurs, accompanied by the release of internal stress and the dissipation of excess free energy, leading to an exothermic reaction detected in the DSC curve. Additionally, residual additives or minute impurities from the electrodeposition process may decompose or redistribute around 476 °C, further contributing to thermal effects. The 615 °C exothermic peak corresponds to significant grain growth and phase stabilization, where the nanocrystalline structure transitions into a more stable FCC (Face-centered cubic) Ni phase, accompanied by heat release. At this stage, minor oxidation of Ni may occur, but its impact on the DSC response is expected to be negligible, as the dominant transformation is the progression toward complete microstructural stabilization. Normal grain growth similar to that of bulk Ni was expected to occur after the exothermic peak at approximately 615 °C, and rapid nanocrystal growth and microstructure stabilization were expected to occur above this critical temperature.

**Figure 3:**
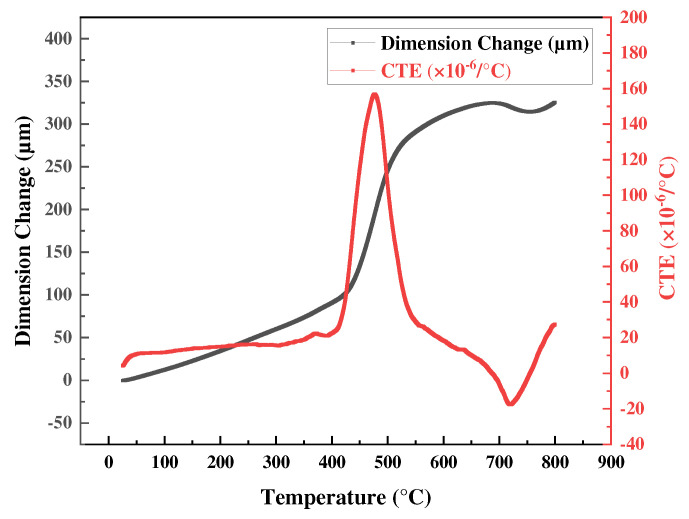
Changes in the dimensional and thermal expansion coefficients of an electrodeposited Ni foil revealed by the film/fiber probe.

The TMA data are shown in Figure 3. A change in linear length was apparent to approximately 400 °C, followed by a sharp increase and then a slower increase after 500 °C. Thermal expansion coefficients (CTEs) were calculated at various points; the CTE increased sharply to 160 × 10^−6^/°C between 400 °C and 600 °C, followed by a decrease, demonstrating the changes in the temperature-dependent material more clearly than the DSC results. In previous studies, the bulk material superplastic deformation temperature was generally 0.5 of the melting temperature (Tm), although, for electrodeposited nanocrystalline Ni, it was reported to be 0.36 Tm [45,46,47]. The TMA data confirmed that nanocrystalline Ni underwent rapid grain growth from the nano- to the micro-scale over a specific temperature range indicated by changes in the CTE, which were dramatic near the superplastic deformation temperature. Thus, superplastic deformation was predicted more accurately by the TMA than by DSC.

### 3.2. Grain Growth and Microstructural Evolution

**Figure 4:**
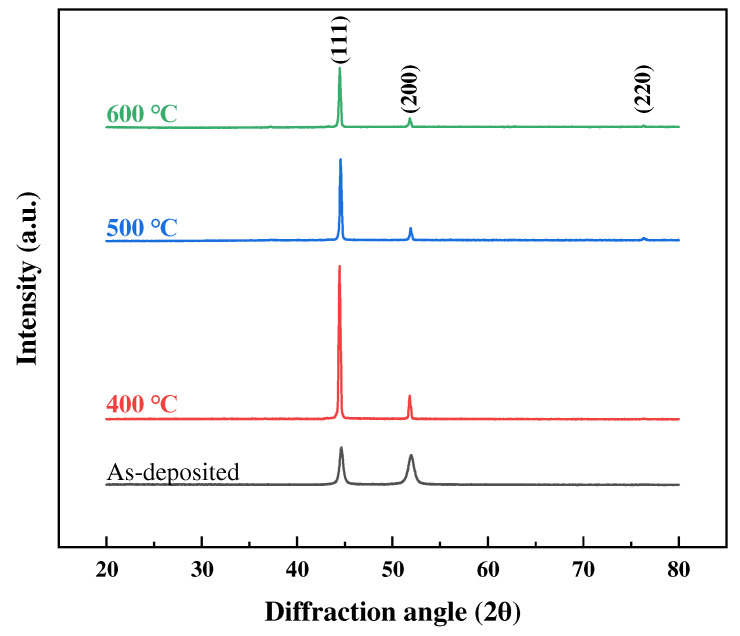
XRD patterns of electrodeposited Ni foils at different annealing temperatures.

Based on the TG-DSC and TMA data for electrodeposited Ni foils, annealing temperatures of 400 °C, 500 °C, and 600 °C were selected. The DSC results indicate that significant microstructural changes occur between 476 °C and 615 °C, which aligns with the critical transformations observed in the TMA analysis. Specifically, the TMA data suggest that a transition influencing the material’s deformation behavior occurs between 400 °C and 600 °C. This temperature range is further supported by previous studies, which have reported that nickel exhibits superplasticity within these conditions [32,33]. In previous studies, electrodeposited Ni coatings exhibited structures similar to those of amorphous materials in the as-deposited state but, after heat treatment, developed a crystalline structure resembling that of bulk Ni [48,49]. The XRD results for Ni foils annealed in a high-purity hydrogen atmosphere are shown in Figure 4. In the as-deposited state, electrodeposited foils exhibited peaks similar to those of amorphous materials, but crystalline structures began to form at temperatures above 400 °C. For all samples, electrodeposited Ni exhibited the strongest diffraction intensity from the (111) plane, but the intensity of the (220) peak increased at higher annealing temperatures. These results are consistent with typical XRD results for electrodeposited Ni foils [50,51].

The grain sizes of Ni foils were derived based on XRD peak broadening, using the Scherrer equation, as follows:

(1) d=(k×λ)/(β ×cos⁡θ)

where *d* represents the crystallite size, k is the Scherrer constant, λ is the wavelength of the X-ray beam, β is the full width at half maximum (FWHM) of the diffraction peak, and *θ* is the Bragg angle. The grain sizes calculated using this equation are presented in Table 2. The grain size of the as-deposited alloy was approximately 20 nm; grain growth was apparent after annealing, as demonstrated by diffraction peak narrowing. However, the grain sizes of samples annealed above 400 °C did not exhibit increases that were proportionate to the temperature because the Scherrer equation does not reliably calculate grain sizes above 100 nm [52,53]. As reported in previous studies on electrodeposited nickel [54], grains in these materials have been shown to grow beyond 100 nm during annealing. However, due to the inherent limitations of the Scherrer equation, the values obtained from XRD analysis remained below 100 nm. Because diffraction peak narrowing may introduce errors, EBSD was used to assess grain growth at the various annealing temperatures more accurately. Using this approach, detailed grain size evolution and microstructural change data were obtained.

**Figure 5:**
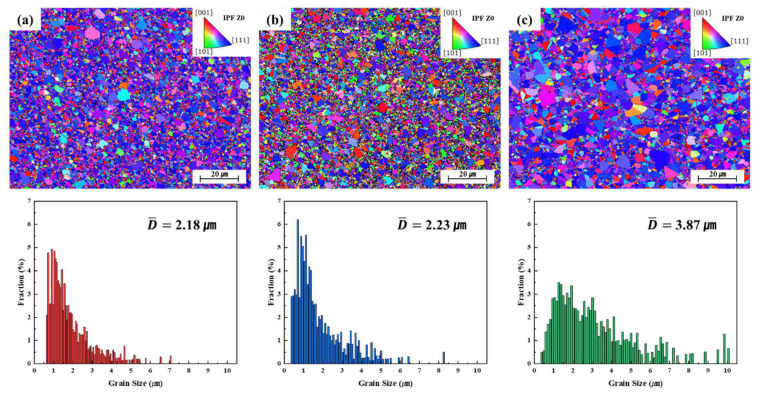
EBSD results from the IPF maps and the grain size distribution histogram of electrodeposited Ni after annealing at (**a**) 400 °C, (**b**) 500 °C, and (**c**) 600 °C.

Figure 5 shows the EBSD data obtained at annealing temperatures of 400 °C, 500 °C, and 600 °C, respectively, over a period of 30 min. As the temperature increased, nanocrystalline grains grew rapidly to the micrometer range. According to previous studies [55,56], as-deposited Ni foils exhibit a nanocrystalline structure. X-ray diffraction (XRD) analysis in this study also confirmed that the as-deposited foils retained their nanocrystalline characteristics. Inverse pole figure (IPF-Z) images of samples annealed at each temperature show that abnormal grain growth (to more than double the average grain size) was locally apparent at 400 °C and 500 °C, with significant coarsening. This may be because the low thickness of the electrodeposited Ni foil contributed to abnormal grain growth. While reduced grain boundary mobility can limit conventional grain growth, alternative mechanisms, such as surface diffusion and stress-assisted grain growth, may become significant in ultrathin films. These effects can promote localized grain coarsening, particularly under impurity segregation or residual stress [57,58]. In particular, the texture of the sample annealed at 500 °C appeared to be random. In contrast, the sample annealed at 600 °C exhibited many grains larger than a few micrometers and a relatively uniform recrystallized structure. EBSD revealed that the average grain sizes of samples annealed for 30 min at 400 °C, 500 °C, and 600 °C were 2.18, 2.23, and 3.87 μm, respectively. Sub-micron grain growth was apparent in the sample annealed at 500 °C; the proportion of sub-micron grains decreased significantly at 600 °C and that of micrometer-scale grains increased. The maximum coarse grain size in annealed samples was approximately 10 μm, thus approaching the foil thickness (10 μm). This may limit grain boundary sliding, which is a primary feature of superplastic deformation. Thus, annealing accelerated grain coarsening.

### 3.3. Superplasticity Analysis Using TMA

**Figure 6:**
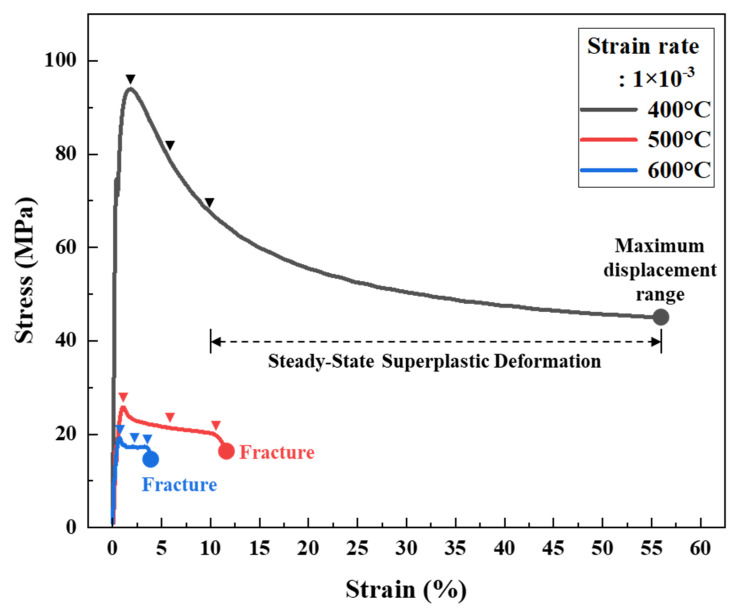
TMA-measured elongation of tensile specimens at a strain rate of 1 × 10^−3^ s^−1^ at 400 °C, 500 °C, and 600 °C.

The tensile test mode of a TMA (maximum load, 1.0 N) is generally used to evaluate low-strength polymer films. However, a stress of 100 MPa can be applied to a metal sheet of thickness 10 μm and width 1 mm, which is a lower stress level than that required for the superplastic deformation of bulk metals. The grain size required for superplastic deformation of bulk materials is typically less than 10 μm, and the strain rate for superplastic deformation is typically 10^−1^ to 10^−5^ s^−1^ [59,60,61]. The strain rate was selected by reference to previous studies that used strain to induce superplastic deformation. In most cases, after annealing, the grain size remained less than 10 μm from 400 °C to 600 °C. To analyze the superplastic properties of Ni foils, samples with a thickness of 10 μm, width of 1 mm, and length of 8 mm were prepared. TMA analysis was performed at 400 °C, 500 °C, and 600 °C, attained at a heating rate of 5 °C/min; the temperature was held for 30 min and deformation occurred at a strain rate of 1 × 10^−3^ s^−1^. The stress-strain curves obtained at each temperature are shown in Figure 6.

At 400 °C, the sample exhibited elongation in excess of 50% without fracture. The stress-strain curve was similar to that of bulk material superplastic deformation revealed by a UTM [29,32]. However, the strain never exceeded 55% because this strain level is associated with a maximum TMA displacement. At 500 °C and 600 °C, the samples fractured at a maximum stress of approximately 20 MPa, and elongation was low (~10%). Superplastic characteristics were apparent near the temperature at which the CTE of electrodeposited Ni foil sharply increased (Figure 3). The Tg measured by DSC was almost identical to that at the maximum CTE rise, but no superplastic deformation was observed beyond Tg. The loss of superplasticity at 500 °C and 600 °C is attributed to both grain growth and deformation mechanism transition. EBSD analysis shows significant grain coarsening (3.17 μm at 500 °C, 5.75 μm at 600 °C; Figure 5), restricting grain boundary sliding and enhancing grain boundary migration. The presence of annealing twins further supports this transition. Superplastic deformation is characterized by high elongation at low stress and creep deformation (a gradual increase in deformation) under sustained stress. At temperatures above Tg, grain growth may be accelerated, potentially restricting grain boundary sliding. Consequently, the deformation mechanism may then partially shift to creep deformation, resulting in early fracture after an initial increase in elongation under low stress. In particular, at temperatures above Tg, deformation mechanisms such as dislocation creep and grain boundary creep may be the predominant contributors to deformation [62,63].

**Figure 7:**
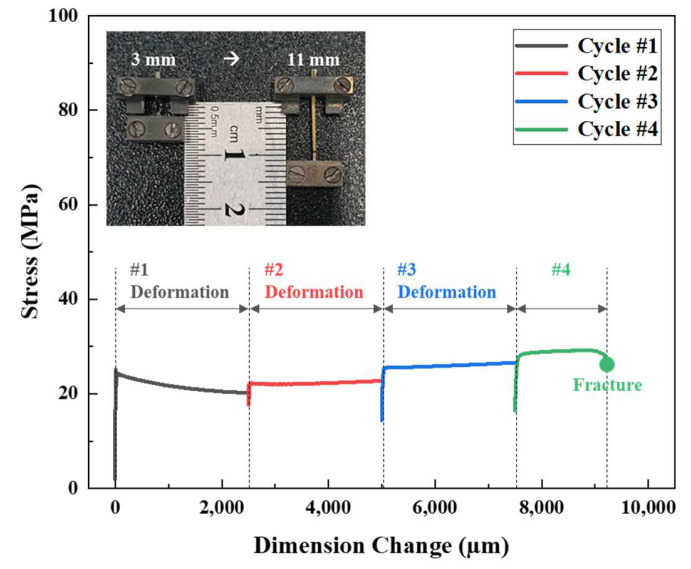
The stress-strain curve and failure of a tensile specimen subjected to TMA testing at a strain rate of 1 × 10^−3^ s^−1^ at 400 °C.

The maximum elongation could not be determined because it exceeded the maximum displacement measured by the TMA. The maximum TMA measurement range is 3.2 mm (the travel range). When displacement increases rapidly, that displacement may exceed the equipment specifications, rendering reliable measurements impossible. Based on previous experimental results, stable measurements were feasible within a displacement of 2.5 mm, and this limitation was considered when designing the experiment. However, a single 2.5 mm displacement did not yield the typically reported elongation of 200–300%. Thus, we used repeated deformations to accumulate the total elongation. Therefore, the method was customized to perform maximum elongation testing of electrodeposited Ni foils. To analyze the elongation-to-failure behavior of Ni foils, samples of 10 μm thickness, 1 mm width, and 3 mm length were prepared. Under the same conditions used in the temperature-specific superplastic experiments, the temperature was increased to 400 °C at a heating rate of 5 °C/min and held for 30 min, followed by deformation at a strain rate of 1 × 10^−3^ s^−1^, with a maximum displacement of 2.5 mm/cycle. After deformation attained approximately 2.5 mm, the program was repeated to fracture (during the fourth cycle). The combined stress-strain curves for each cycle are shown in Figure 7; the maximum elongation was approximately 306%. The increase in yield strength during the subsequent deformation cycles surpassed the flow stress of the previous cycles. This is not readily explained by the inherent mechanical properties of the material alone. Rather, such behavior likely reflects the displacement control mechanism of the TMA. Given the short inter-cycle dwell time prior to the next deformation cycle, residual plastic deformation may induce localized concentrations of stress, thereby amplifying the initial stress response. This may contribute to the nonlinear stress behavior observed during the TMA experiments.

Traditional UTM-based analysis has revealed a maximum electrodeposited Ni foil elongation of 2520% under the same deformation conditions [33]. This large difference may be attributable to variations in the plating bath conditions, strain rate, or deformation atmosphere. In addition, the small cross-sectional areas of our samples compared to those subjected to UTM analysis increased the likelihood of fracture when small voids formed during deformation. Therefore, it may be difficult to determine the absolute maximum deformation of an electrodeposited metal foil using a TMA; however, a TMA remains very useful in that it reveals the development of superplastic characteristics under specific conditions.

**Figure 8:**
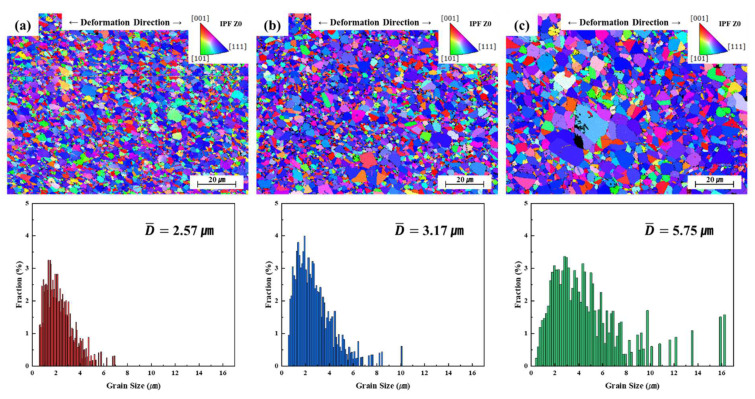
The EBSD maps and the grain size distribution histogram of fractured tensile samples at (**a**) 400 °C, (**b**) 500 °C, and (**c**) 600 °C.

The microstructures revealed by the TMA near the fractures of samples deformed at 400 °C, 500 °C, and 600 °C at a strain rate of 1 × 10^−3^ s^−1^ are shown in Figure 8. The shapes of grains that were superplastically deformed differed significantly from those of bulk microstructures treated at the same temperatures (Figure 5). At 400 °C, a sample that underwent superplastic deformation maintained an average grain size of 2 μm, similar to the heat-treated sample shown in Figure 5. Because the equiaxed grain shape was retained after deformation, grain boundary sliding was probably the primary deformation mechanism. Although the average grain size was similar to that shown in Figure 5a, uniform grain growth was observed across all regions, unlike the partial grain growth observed in the heat-treated sample. As the deformation temperature increased, the grain size rose compared to those shown in Figure 5b,c. The average grain sizes after deformation at 500 °C and 600 °C were 3.17 μm and 5.75 μm, respectively; grain growth was accelerated by external stress at higher temperatures. Although the 600 °C sample exhibited no superplastic deformation, grain growth to sub-micron or micron levels during deformation facilitated dislocation movement, and voids and cavities formed and grew. This suggests that superplastic deformation may have been inhibited because grain boundary sliding was restricted as the grain size approached the foil thickness. Excessive grain growth may render intragranular deformation more common than grain boundary deformation, associated with deformation behavior more similar to conventional high-temperature creep deformation than superplastic deformation [64,65]. Such a shift in the deformation mechanism may be a primary reason why superplasticity was not observed at 600 °C.

**Figure 9:**
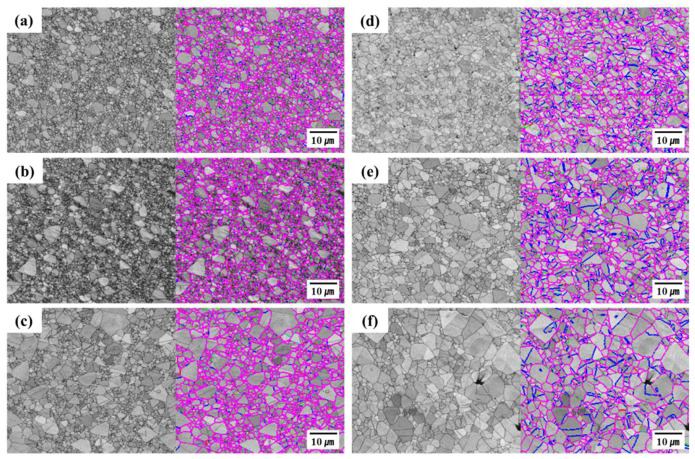
SEM micrographs and image quality maps of electrodeposited Ni foils annealed at 400 °C, 500 °C, and 600 °C, respectively (**a**–**c**), and after superplastic deformation at the same temperatures at a strain rate of 1 × 10^−3^ s^−1^ (**d**–**f**).

Figure 9 shows SEM micrographs and image quality maps of electrodeposited Ni foils annealed and superplastically deformed at different temperatures. Figure 9a–c correspond to samples annealed at 400 °C, 500 °C, and 600 °C without strain, respectively, and Figure 9d–f correspond to samples deformed at the same temperatures at a strain rate of 1 × 10^−3^ s^−1^. The SEM images reveal distinct twin structures predominantly within grains but fewer twins near the grain boundaries of both the annealed and superplastically deformed samples. The formation and distribution of such twin structures exhibited similar patterns in both the annealed and deformed samples, suggesting that the crystallographic texture of electrodeposited nanocrystalline Ni foils may be attributable to twin formation during both annealing and deformation. In the annealed samples, equiaxed grains lacking a distinct texture appeared at 400 °C, and annealing twins were observed within grains at 500 °C and 600 °C. This suggests that grain boundary migration during annealing was involved in the formation of the observed microstructures. The presence of twin structures in both the annealed and deformed samples indicates that the crystallographic texture of electrodeposited Ni foils formed via both grain boundary migration and twin boundary rearrangement.

The superplastic deformation of electrodeposited nanocrystalline Ni foils is associated with ultrafine grains in the nanocrystalline structure. Such a fine-grained structure facilitates grain boundary sliding even at relatively low temperatures. The stable twin boundaries observed during both annealing and deformation may allow relative grain boundary displacement, thereby contributing to superplastic deformation. Such microstructural characteristics provide visual evidence supporting the hypothesis that electrodeposited Ni foils exhibit superplastic deformation at lower temperatures than bulk materials. Bulk superplastic deformation generally requires temperatures higher than half of the melting temperature, but not for electrodeposited nickel samples, in which superplastic deformation occurs below 35% of the melting temperature. Such superplastic deformation at lower temperatures greatly increases processing efficiency compared to bulk materials.

## 4. Conclusions

We analyzed electrodeposited nanocrystalline Ni foils of 1 mm width and 10 μ thickness m. Using TG-DSC and a TMA, we predicted the temperature range over which superplastic deformation might occur and used EBSD to observe superplastic deformation and microstructural changes. Electrodeposited nanocrystalline Ni foils exhibited rapid thermal behavioral changes between 400 °C and 600 °C, with significant grain growth at the micrometer scale along a (preferred) (111) orientation. Microstructural observations at various temperatures revealed that as the temperature increased from 400 °C to 600 °C, grain growth developed. When stress was applied to trigger superplastic deformation under the same conditions, grain growth became more rapid. The electrodeposited Ni foils exhibited a maximum elongation of 306% at 400 °C under a strain rate of 1 × 10^−3^ s^−1^, with equiaxed grain structures maintained after deformation. Superplasticity was absent at 500 °C and 600 °C. TMA thermal analysis indicated that superplasticity can occur in the region where the CTE sharply increases. Furthermore, the superplastic behavior of thin metal foils can be directly measured using a TMA. This is an effective alternative to conventional UTM methods when analyzing ultrathin samples.

Together, these findings demonstrate that a TMA is a useful and novel tool for exploring the superplastic characteristics of metal foils less than 10 μm in thickness.

## Figures and Tables

**Table 1:** Bath composition and the electrodeposition parameters.

Electrodeposition Parameters	Value	
Electrolyte composition (g/L)	Ni(SO_3_NH_2_)_2_·4H_2_O	180
	NiCl_2_·6H_2_O	20
	H_3_BO_3_	30
	C_7_H_5_NO_3_S	1
	CH_3_(CH_2_)_11_OSO_3_Na	0.1
Temperature (°C)	50	
pH	4	
Current density (mA/cm^2^)	60	
Cathode/Anode	Stainless steel 304L/Nickel S-Rounds in Ti basket	
Agitation	Paddle agitator, Overflow circulation	

**Table 2:** Grain sizes of as-deposited and annealed Ni foils.

Condition	Phase Analysis	2 *θ* (Deg)	FWHM	Grain Size (nm)
As-deposited	FCC (111)	44.596	0.412	21.78
	FCC (200)	51.907	0.699	13.21
	FCC (220)	76.570	0.400	26.45
Annealed at 400 °C	FCC (111)	44.432	0.157	57.31
	FCC (200)	51.790	0.150	61.54
	FCC (220)	76.280	0.140	75.41
Annealed at 500 °C	FCC (111)	44.548	0.155	57.97
	FCC (200)	51.905	0.167	55.30
	FCC (220)	76.349	0.194	54.45
Annealed at 600 °C	FCC (111)	44.454	0.153	58.55
	FCC (200)	51.799	0.155	59.55
	FCC (220)	76.300	0.161	65.59

## Data Availability

The data presented in this study are available on request from the corresponding author due to privacy.
